# Detecting Aortic Valve Anomaly From Induced Murmurs: Insights From Computational Hemodynamic Models

**DOI:** 10.3389/fphys.2021.734224

**Published:** 2021-10-06

**Authors:** Shantanu Bailoor, Jung-Hee Seo, Stefano Schena, Rajat Mittal

**Affiliations:** ^1^Department of Mechanical Engineering, The Johns Hopkins University, Baltimore, MD, United States; ^2^Division of Cardiac Surgery, Johns Hopkins Medical Institute, Baltimore, MD, United States

**Keywords:** aortic valve murmurs, TAVR, computational fluid dynamics, hemoacoustics, supervised learning, anomaly detection

## Abstract

Patients who receive transcatheter aortic valve replacement are at risk for leaflet thrombosis-related complications, and can benefit from continuous, longitudinal monitoring of the prosthesis. Conventional angiography modalities are expensive, hospital-centric and either invasive or employ potentially nephrotoxic contrast agents, which preclude their routine use. Heart sounds have been long recognized to contain valuable information about individual valve function, but the skill of auscultation is in decline due to its heavy reliance on the physician’s proficiency leading to poor diagnostic repeatability. This subjectivity in diagnosis can be alleviated using machine learning techniques for anomaly detection. We present a computational and data-driven proof-of-concept analysis of a novel, auscultation-based technique for monitoring aortic valve, which is practical, non-invasive, and non-toxic. However, the underlying mechanisms leading to physiological and pathological heart sounds are not well-understood, which hinders development of such a technique. We first address this by performing direct numerical simulations of the complex interactions between turbulent blood flow in a canonical ascending aorta model and dynamic valve motion in 29 cases with healthy and stenotic valves. Using the turbulent pressure fluctuations on the aorta lumen boundary, we model the propagation of heart sounds, as elastic waves, through the patient’s thorax. The heart sound may be recorded on the epidermal surface using a stethoscope/phonocardiograph. This approach allows us to correlate instantaneous hemodynamic phenomena and valve motion with the acoustic response. From this dataset we extract “acoustic signatures” of healthy and stenotic valves based on principal components of the recorded sound. These signatures are used to train a linear discriminant classifier by maximizing correlation between recorded heart sounds and valve status. We demonstrate that this classifier is capable of accurate prospective detection of anomalous valve function and that the principal component-based signatures capture prominent audible features of heart sounds, which have been historically used by physicians for diagnosis. Further development of such technology can enable inexpensive, safe and patient-centric at-home monitoring, and can extend beyond transcatheter valves to surgical as well as native valves.

## Introduction

Heart diseases are the leading cause of death in the United States, with more than 600,000 associated annual deaths (Kung et al., 2008). Dysfunctions of the aortic valve are significant contributors to the total heart disease burden of which aortic stenosis (AS) is the most prevalent, affecting approximately 7% of the population (Arora et al., 2017). Often valve repair is not possible and the only recourse for patients is a complete valve replacement. Historically, the gold standard of aortic valve replacement has been surgical replacement (SAVR), but it is limited to patients who can handle the trauma of an open-heart surgery. In the last two decades, transcatheter aortic valve replacement (TAVR) has emerged as a viable AVR modality, especially for frail and high-risk patients. TAVR is minimally invasive, requires relatively mild sedation and the patient can be discharged within 48 h of implantation. Post-implant patient management is also simplified with TAVR, as recipients typically do not require lifelong anticoagulant therapy. Through improvements in valve design, fabrication, and delivery techniques, mortality risk reduction with TAVR has been shown to be non-inferior to that with SAVR. Due to comparable patient outcomes and a significant reduction in periprocedural trauma, TAVR has recently been expanded to include moderate- and low-risk patients. The bioprosthetic valves (BPV) employed in TAVR are made of tissue grafted from porcine or bovine pericardium, which are less thrombogenic and have overall better hemodynamic characteristics, compared to mechanical valves, even in low- and moderate-risk patients with severe AS (Reardon et al., 2017; Kolte et al., 2019).

However, these BPVs are less durable compared to mechanical valves and degenerate within 15 years of implantation, often due to leaflet thrombosis, paravalvular leaks (Généreux et al., 2013), leaflet tears (Gurvitch et al., 2011) or infective endocarditis (Amat-Santos et al., 2015). Of these, leaflet thrombosis can result in reduced leaflet motion (RLM), causing abnormal transvalvular gradients and poor hemodynamic patterns. While its onset may be asymptomatic, it can progress to clinical valve thrombosis and increase the risk herat failure or thromboembolic events (Makkar et al., 2015; Chakravarty et al., 2017). Thus, leaflet thrombosis poses a serious threat to the patient, increasing both morbidity and mortality. Incidence of early leaflet thrombosis (ELT) has been seen in most TAVs on the market (De Marchena et al., 2015; Makkar et al., 2015; Chakravarty et al., 2017) and the exact mechanisms behind the progression/resolution of ELT remain unknown (Rosseel et al., 2019). Therefore, there is an unmet need for longitudinal monitoring of implanted valve, which can enable proactive ELT detection.

Techniques commonly implemented in cardiac imaging, such as transesophageal echo or 4D multidetector CT are expensive, hospital-centric, may employ nephrotoxic contrast-enhancing agents (Andreucci et al., 2014) and are either invasive or radiative. All these issues preclude their routine use and ELT is only incidentally detected during scheduled follow-up examinations (Rosseel et al., 2019). Thus, TAVR recipients can benefit from a novel, longitudinal monitoring modality which is patient-centric, inexpensive, non-invasive, and non-toxic. Recent strides in sensor-miniaturization, smart materials, telemetry, machine learning have led to the proliferation of wearable, implantable and embedded biosensor-based health monitoring devices. While limited inroads have been made into sensor-based monitoring techniques for cardiac health (Fonseca et al., 2006; Chen et al., 2014; Hermans et al., 2018), very few developments address the needs of TAVR patients. For instance, proof-of-concept analyses of sensorized mechanical (Marcelli et al., 2018) and transcatheter (Bailoor et al., 2021b) aortic valves have been recently proposed, but these devices cannot assist patients with existing TAVs. In this investigation, we seek to develop a safe and practical valve monitoring modality which can be independently used by patients with existing and future implants.

We employ cardiac auscultation, a safe, centuries-old diagnostic technique, as the basis for our proposed modality. Blood flow associated with many heart diseases, and particularly heart valve diseases, generate characteristic sounds, called “murmurs,” which can be accurately interpreted by a well-trained physician. The technique is inexpensive, non-invasive, and can be safely performed at home. Despite these advantages, this seemingly essential skill has been in decline for the past few decades (Alam et al., 2010), mainly due to its heavy reliance on the physician’s acuity and proficiency. Studies and surveys have shown that this decline is attributed to the lack of auscultation training given to medical students and poor improvement in their diagnostic accuracy following subsequent training (Dhuper et al., 2007). These issues hamper repeatability of diagnosis. Studies have also shown that low diagnostic accuracy of manual auscultation is an international issue (Mangione, 2001). The advent of digital stethoscopes facilitates noise-cancelation, signal amplification and analysis to assist physicians. The dependence of cardiac auscultation on the subjective judgment of a physician can be considerably reduced for better diagnostic repeatability by integrating modern sensing technology with machine learning techniques for pattern recognition and anomaly detection.

Another issue with auscultation is that the underlying causal mechanisms which lead to heart sounds are not well-understood. This issue is compounded by inherent limitations of performing simultaneous measurements of heart murmurs and the underlying hemodynamics, due to which, *in vivo* and *in vitro* experiments can only use very simple models. Some recent studies (Dominguez-Morales et al., 2018; Chorba et al., 2021) performed *post-hoc* analyses on human phonocardiographic measurements using neural networks to detect different types of heart valve diseases. However, they provide little description of the underlying physical phenomena or their correlation with pathological heart sounds. *In silico* modeling seems more promising, in that there are no restrictions on the type and number of concurrent measurements, and it is easy to use in conjunction with machine learning. Some computational studies have been performed on wave propagation in tissue-like materials (Yazicioglu et al., 2005; Ozer et al., 2007), but they used prescribed sound sources as opposed to coupling with physiological blood flow. Recently Seo et al. developed a computational hemoacoustic (CHA) solver (Seo et al., 2017) to study the generation and propagation of flow-induced murmurs through tissue-like media and showed how stenotic aortas, modeled as circular tubes with static constrictions, can result in detectable sound when sound generation is coupled with hemodynamics. The authors, however, do not account for dynamic valve motion or the effects of flow pulsatility which influence transient blood flow patterns in the aorta.

Through this investigation, we provide the first of its kind description of physiologically realistic fluid-structure interaction (FSI) inside the ascending aorta with healthy and stenotic TAVs and the consequent sound generation and propagation in the surrounding tissue-like material. Further, we present a proof-of-concept analysis of a novel, data-driven, inexpensive, and safe method to process such heart sounds recorded on a patient’s thorax and subsequently infer the health of the valve. The paper is organized as follows: In section 2, we present numerical methods used to simulate blood flow inside the aorta, the resulting aortic valve motion, and sound propagation in a homogenous medium. Then in section 3, we use direct simulation to describe differences in blood flow patterns and the corresponding heart sounds resulting from healthy and stenotic TAVs, as recorded on at the aortic valve port. Next, a data-driven methodology is developed on a dataset of such simulations to extract statistically significant differences between physiological and pathological heart sounds. These differences are used to train a machine learning algorithm to detect early leaflet thrombosis induced aortic stenosis. The trained algorithm is tested on a new set of simulations to test its predictive ability. In section 4, we discuss important temporal features of the acoustic signals which are most relevant to classification. Finally, in section 5 we present concluding remarks and future of this research.

## Materials and Methods

### Immersed Boundary Flow Solver

The work described in this manuscript builds upon methods described in previous studies from our group (Mittal et al., 2008; Seo et al., 2017, 2020; Bailoor et al., 2021a,b). The three-dimensional, incompressible Navier-Stokes equations (1) are integrated on a Cartesian grid, finite-difference based flow solver:


$$\nabla \cdot \vec{U}=0$$

(1) $$\rho_{0}\,\left[\frac{\partial \vec{U}}{\partial t}+\left(\vec{U}\cdot \nabla \right)\,\vec{U}\right]=-\nabla P+\mu_{0}\,\nabla^{2}\vec{U}$$

In the above equation, $\vec{U}=\left(U,V,W\right)$ denotes blood flow velocity vector and *P* represent its local pressure. Blood density ρ_0_ and dynamic viscosity μ_0_ are set to 1,060 *kg*/*m*^3^ and 4*mPa*⋅*s*, respectively. The immersed boundary is discretized using triangular surface elements and the fluid-solid coupling is handled by a sharp interface immersed boundary method (Mittal et al., 2008). This solver has been extensively validated and shown to accurately describe flow physics in a wide array of cardiovascular applications, ranging from left ventricles (Seo and Mittal, 2013; Vedula et al., 2014), aortas (Zhu et al., 2018; Seo et al., 2020; Bailoor et al., 2021a), and coronary arteries (Zhu et al., 2019). The interested reader is directed to these references for details of this solver.

### Aorta and Aortic Valve Model

**FIGURE 1:**
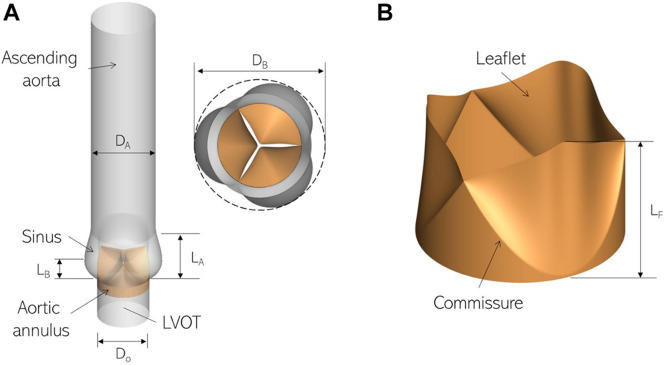
Three-dimensional models labeling important parts and dimensions for **(A)** the ascending aorta and **(B)** aortic valve employed in this study.

We employ a canonical ascending aorta model, which includes the left ventricular outflow tract (LVOT), the aortic annulus and sinus, as shown in Figure 1A. The aortic valve is comprised of three leaflets which are modeled as zero-thickness membranes (Figure 1B). The cross-sections of the LVOT, annulus and aorta are assumed to be smooth and circular and important dimensions are based on anatomic measurements (Reul et al., 1990), listed in Table 1.

**TABLE 1:** Important dimensions in our aorta and aortic valve models.

**Component**	**Measurement**	**Value (mm)**
LVOT	Diameter (Do)	23.00
Aortic Root	Root Diameter (DB)	35.65
	Axial Length (L_A_)	23.00
	Base Length (L_B_)	7.82
Ascending Aorta	Diameter (D_A_)	28.50
Aortic Valve	Leaflet Length (L_f_)	15.00

In the next section, we describe how forces from the fluid subsystem are used to drive valve leaflet motion through reduced degree-of-freedom dynamics.

### Reduced Degree-of-Freedom Valve Dynamics

**FIGURE 2:**
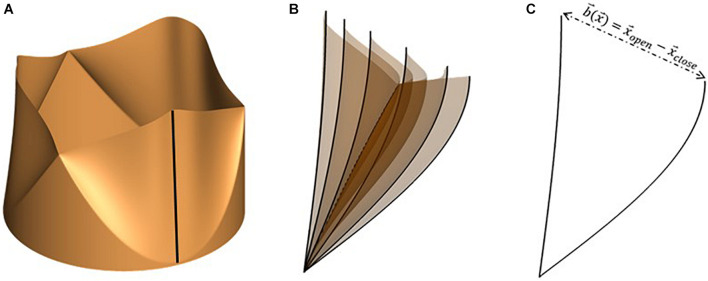
**(A)** Idealized valve model with circular cross-section with leaflet centerline highlighted using a black line, **(B)** snapshots of leaflet centerline at several instances during valve opening and **(C)** leaflet centerline at maximally opened and closed configurations, indicating the local range-of-motion vector, $\vec{\text{b}}$$\left(\vec{\text{x}}\right)$.


We employ a versatile reduced degree-of-freedom (rDOF) valve model to facilitate generating efficient, high-fidelity simulations for patient-specific anatomies. Beginning from an idealized model with circular cross-section (Figure 2A), we use simple mathematical transformations to adapt it to individual patient annulus morphology. Since we are more interested in an accurate description of transvalvular and ascending aorta hemodynamics compared to stress distributions in the valve leaflets, some simplifications in the aortic valve model can be made, if important kinematic features of valve motion are incorporated. Therefore, leaflet motion in our valve model follows a simple equation of motion (Equation 2), shown below:

(2) $$\alpha \,\frac{\partial {\vec{v}}_{v}}{\partial t}=△\,p\,\vec{n}-\kappa \,\left({\vec{d}}_{v}-{\vec{d}}_{v,0}\right)$$

where, ${\vec{d}}_{v}$ and ${\vec{v}}_{v}$ represent instantaneous leaflet position and velocity, respectively, and ${\vec{d}}_{v,0}$ is the fully closed leaflet configuration. The valve model is parametrized using two constants (α,κ), relating to leaflet mass and stiffness, such that equation (2) describes a balance between leaflet inertia (α), driving pressure difference across leaflet surface (△*p*), and restoring forces due to linear tissue elasticity (κ). Instantaneous leaflet displacement is expressed in terms of a range-of-motion vector, $\vec{b}\,\left(\vec{x}\right)$, and a mapping function $\xi \,\left(\vec{x},c\,\left(t\right)\right)$, as shown in equation (3).


(3) $${\vec{d}}_{v}\,\left(\vec{x},t\right)=\vec{b}\,\left(\vec{x}\right)\,\xi \,\left(\vec{x},t\right)$$

The range-of-motion vector is defined for each point on the leaflet surface as the difference between its coordinates at maximally open and closed configurations (equation 4). An illustration of the same is shown in Figure 2 for the free end of one leaflet centerline (shown using the dark black line in Figure 2A). The motion of the centerline over several phases during valve opening is shown in Figure 2B. From these, the fully open and closed leaflet configurations are isolated in Figure 2C, indicating the vector $\vec{b}\,\left(\vec{x}\right)$ for its free edge.


(4) $$\vec{b}\,\left(\vec{x}\right)={\vec{x}}_{o\,p\,e\,n}-{\vec{x}}_{c\,l\,o\,s\,e}$$

**FIGURE 3:**
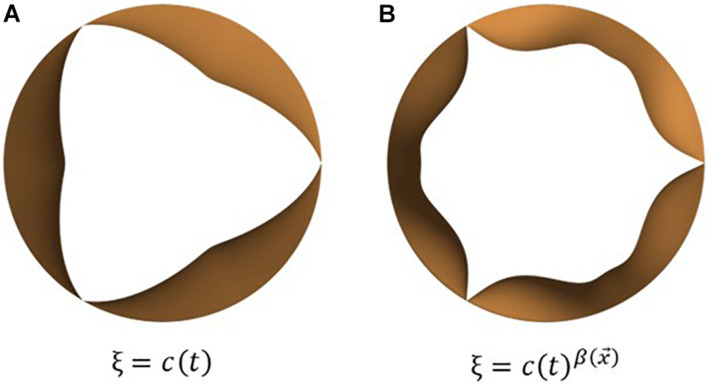
Examples of leaflet kinematic features which can be obtained by tuning the mapping function, ξ: commonly observed opening mode shapes can be obtained using simple mathemtical dependencies such as **(A)** linear and **(B)** power relations between the mapping function and lumped displacement.


The mapping function ξ is scalar-valued and depends on the location on the leaflet surface and the instantaneous phase in the cardiac cycle, via a lumped displacement *c*(*t*) [0 < *c*(*t*) < 1]. The purpose of the mapping function is to compute the instantaneous leaflet displacement via interpolation on the range of motion vector using the lumped displacement and appropriate distribution of the same over the leaflet surface to obtain desired leaflet kinematic features. Here, ξ describes the spatio-temporal dependence of opening/closing motion of individual leaflets and thus governs leaflet opening/closing mode shapes. To replicate commonly observed valve shapes, we tested two mapping functions, one depending linearly, and the other through a power-law relation, on *c*(*t*), as seen in equations (5) (a) and (b), respectively. The corresponding leaflet mode shapes are illustrated in Figures 3A,B.

(5a) $$\xi \,\left(\vec{x},t\right)=c\,\left(t\right)$$

(5b) $$\xi \,\left(\vec{x},t\right)=c\,{\left(t\right)}^{\beta \,\left(\vec{x}\right)}$$

Differentiating the ansatz for leaflet position we get an expression for the local velocity, as shown in equation (6).


(6) $${\vec{v}}_{v}\,\left(\vec{x},t\right)=\frac{d\,c}{d\,t}\,\left(t\right)\,\frac{\partial \xi }{\partial c}\,\left(\vec{x},c\,\left(t\right)\right)\cdot \vec{b}\,\left(\vec{x}\right)$$

Next, we substitute the displacement and velocity ansatz in the normal component of the leaflet equation of motion (equation 2) and integrate over leaflet surface to obtain the following second-order ODE for the lumped displacement:


$$\frac{d^{2}\,c}{d\,t^{2}}=\frac{F_{P}-F_{S}-F_{m,V}}{\alpha \,\int \left(\frac{\partial \xi }{\partial c}\,\vec{b}\cdot \vec{n}\,d\,s\right)},\text{where}$$

$$F_{P}=\int \Delta \,p\,ds$$

$$F_{S}=\int \kappa \,\left(\xi \,\left(\vec{x},t\right)-\xi \,\left(\vec{x},0\right)\right)\,\vec{b}\cdot \vec{n}\,ds$$

(7) $$F_{m,V}=\alpha \,\int \frac{\partial^{2}\xi }{\partial c^{2}}\,{\left(\frac{d\,c}{d\,t}\right)}^{2}\,\vec{b}\cdot \vec{n}\,ds$$

**FIGURE 4:**
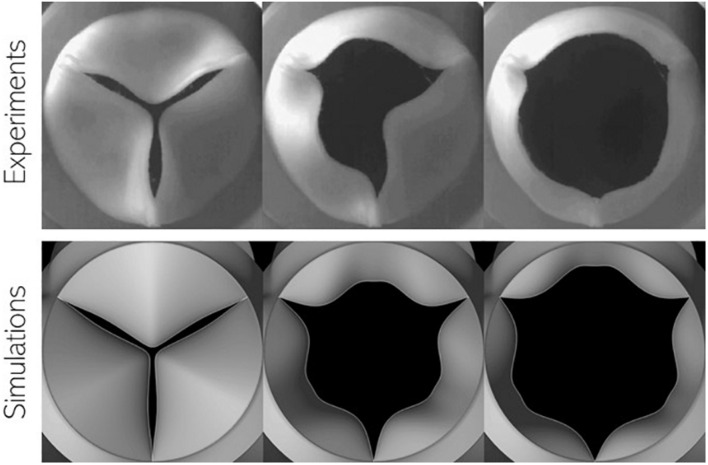
Comparison of valve configuration at three instances during leaflet opening using (top) high-speed imaging of *in vitro* experiments using bioprosthetic valves and (bottom) present simulations.

In the above equation, *F*_*P*_, *F*_*S*_, and *F*_*m,V*_ represent forces due to pressure difference across the leaflet surface, restoring forces due to tissue elasticity and inertial effects arising from non-linear mapping functions, respectively. This model is capable of mimicking observed bioprosthetic valve kinematics in terms of opening shape as observed in Figure 4: we compare snapshots of a valve with power mapping during three instants in opening with corresponding valve configurations from high-speed imaging of bioprosthetic valves (courtesy L.P. Dasi). The valve leaflets show similar features in each configuration indicating that the mapping function can be successfully tuned to achieve desired valve shapes. The model, however, does not account for more complex features, such as the sticking of valve leaflets (middle).

In addition to matching bioprosthetic valve kinematics, the dynamics of its motion can also be accurately captured by appropriate tuning of the model parameters (α,κ). For healthy valve leaflets, leaflet inertia α=40*kg*/*m*^2^ and stiffness κ=10,600*Pa*/*m*. These parameters were determined by matching the evolution of projected valve open area with predictions from a detailed 3D nonlinear finite element model (de Tullio and Pascazio, 2016; Bailoor et al., 2021a). Pathological valve function in terms of aortic stenosis can be easily modeled by increasing the value of leaflet stiffness (κ) or inertia (α) parameters over their baseline healthy values. More details about this fluid-structure-interaction solver, including solution verification, valve model tuning and validation can be found in our earlier work (Bailoor et al., 2021a,b).

### Murmur Generation and Propagation Model

Heart murmurs are propagated through biological tissue as elastic (compression and shear) waves, which can be modeled using the generalized Hooke’s law with the Kelvin-Voigt viscoelastic model. The corresponding governing equations are shown below:


$$\frac{\partial p_{i\,j}^{\prime }}{\partial t}+\lambda \,\frac{\partial u_{k}^{\prime }}{\partial x_{k}}\,\delta_{i\,j}+\mu \,\left(\frac{\partial u_{i}^{\prime }}{\partial x_{j}}+\frac{\partial u_{j}^{\prime }}{\partial x_{i}}\right)=0$$

(8) $$\rho_{s}\,\frac{\partial u_{i}^{\prime }}{\partial t}+\frac{\partial p_{i\,j}^{\prime }}{\partial x_{j}}=\eta \,\frac{\partial }{\partial x_{j}}\,\left(\frac{\partial u_{i}^{\prime }}{\partial x_{j}}+\frac{\partial u_{j}^{\prime }}{\partial x_{i}}\right)$$

In equation (8), $p_{i\,j}^{\prime }$ and $u_{i}^{\prime }$ represent local elastic stress and velocity fluctuations, respectively. The surrounding organs, bones, blood volume and other tissue are represented by a homogenous medium with density ρ_*s*_, Lamé parameters λ and μ, and viscosity η. Finally, δ_*ij*_ denotes the Kronecker delta. Equation (8) describes the propagation and dissipation of compressive (bulk) and shear waves within a homogenous viscoelastic medium. The tissue-air interface on the epidermis is treated as a traction-free surface and the corresponding boundary condition is:


(9) $$p_{i\,j}^{\prime }\,n_{j}=0,\left[\lambda \,\frac{\partial u_{k}^{\prime }}{\partial x_{k}}\,\delta_{i\,j}+\mu \,\left(\frac{\partial u_{i}^{\prime }}{\partial x_{j}}+\frac{\partial u_{j}^{\prime }}{\partial x_{i}}\right)\right]\,n_{j}=0$$

In the above equation, *n_j* represents the surface normal vector. To simplify the boundary condition on the aorta-surrounding tissue interface, the viscous shear stress induced by blood can be assumed to be negligible compared to normal (pressure) forces. The resulting boundary condition is specified as follows:


(10) $$p_{i\,j}^{\prime }\,n_{j}=P^{\prime }\,n_{i},\left[\lambda \,\frac{\partial u_{k}^{\prime }}{\partial x_{k}}\,\delta_{i\,j}+\mu \,\left(\frac{\partial u_{i}^{\prime }}{\partial x_{j}}+\frac{\partial u_{j}^{\prime }}{\partial x_{i}}\right)\right]\,n_{j}=-\frac{\partial P^{\prime }}{\partial t}$$

In the above equation, $P^{\prime }=P-\bar{P}$ is the hemodynamic pressure fluctuation on the aorta lumen boundary. For the homogenous medium described above, the compression wave speed is given by $c_{B}=\sqrt{K/\rho_{s}}$ where *K* = λ + 2μ/3 represents the bulk modulus. Likewise, the shear wave speed is defined as $c_{S}=\sqrt{\mu /\rho_{s}}$. The compression wave speed calculated above is much larger than the characteristic blood flow velocity in the aorta. Thus, conducting a coupled computational hemoacoustic simulation would necessitate an excessively restrictive value of time-step on the flow-solver. Moreover, the influence of shear and compression waves on blood flow inside the aorta is expected to be negligible. Thus, using a one-way coupling between the FSI and acoustics can be justified, using hemodynamic forcing on the elastic wave propagation simulation. At low frequencies (<1000 Hz), the solution of equation (8) can be approximated by using the free space Green’s tensor in the frequency domain:


$$G_{i\,j}\left(\vec{r},\omega \right)=\frac{i\,k_{p}}{12\,\pi \,\left(\lambda +2\,\mu \right)}(\delta_{i\,j}h_{0}^{\left(1\right)}\left(k_{p}r\right)+\left(\delta_{i\,j}-3\frac{x_{i}\,x_{j}}{r^{2}}\right)$$

$$h_{n}^{\left(1\right)}\left(k_{p}r\right))-\frac{i\,k_{s}}{12\,\pi \,\mu }(-2\delta_{i\,j}h_{0}^{\left(1\right)}\left(k_{s}r\right)+\left(\delta_{i\,j}-3\frac{x_{i}\,x_{j}}{r^{2}}\right)h_{n}^{\left(1\right)}\left(k_{s}r\right))$$

**FIGURE 5:**
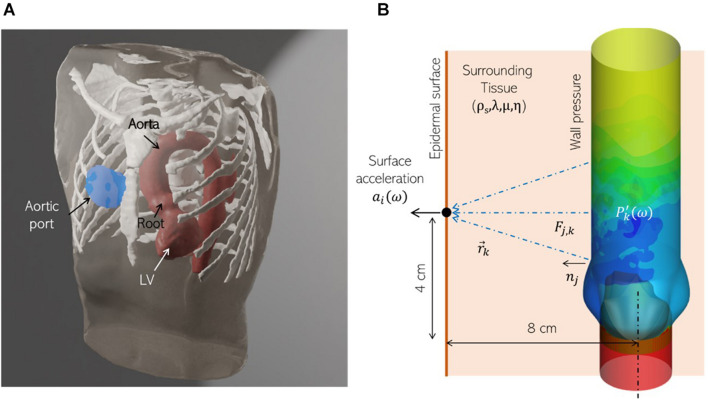
**(A)** A realistic human thorax model comprising of an adult male torso, thoracic skeletal structures (white) and a patient-specific left ventricle (LV) and thoracic aorta (red), showing the auscultation “post” for the aortic valve (blue) at the sternum, near the right second intercostal space. The torso and skeletal anatomy were sourced from the Visible Human Project (An anatomical data set developed under a contract from the National Library of Medicine by the Departments of Cellular and Structural Biology, and Radiology, University of Colorado School of Medicine https://www.nlm.nih.gov/research/visible/visible_human.html, while the LV and aorta model were segmented from a de-identified patient-specific thoracic CT scan. **(B)** Schematic showing the corresponding idealized aorta + thorax model employed for computational hemoacoustic modeling.

In the above equation, $k_{p}=\omega /\sqrt{\left(\lambda +2\,\mu \right)/\rho_{s}}$ is a longitudinal wavenumber, $k_{s}=\omega /\sqrt{\mu /\rho_{s}}$ is a shear wavenumber, $h_{n}^{\left(1\right)}$ is the spherical Hankel function of the first kind, *x_j* is the vector from the source to the monitor point, and *r* is its magnitude. In clinical practice, the aortic valve (AV) is auscultated near the right second intercostal space, near the sternum (Figure 5A). We assume this distance to be 4 cm downstream from, and 8 cm anterior of, the aortic valve. The corresponding location of the monitor point is shown in Figure 5B. We use the Green’s tensor to model elastic wave propagation from pressure force on the aorta surface to this point on the epidermis through a homogenous medium representing surrounding tissue, characterized by tissue parameters: ρ_*s*_, λ, μ, and η. The aorta surface is discretized into 180 surface elements and the surface acceleration recorded at the monitor point is computed as the superposition of the individual contributions from these sources.

Thus, the ‘*n*’ component of surface acceleration is computed as:


(12) $${\hat{a}}_{n}\,\left(\omega \right)=2\,\sum_{k}{\left(-i\,\omega \right)}^{2}\,G_{n\,j}\,\left({\vec{r}}_{k},\omega \right)\,F_{j,k}\,\left(\omega \right)$$

In the above equation, $F_{j,k}\,\left(\omega \right)=n_{j,k}\,{\hat{P}}_{k}\,\left(\omega \right)\,△\,A$, where ${\hat{P}}_{k}\,\left(\omega \right)$ is the hemodynamic pressure on the aorta lumen boundary in the frequency domain from the *k*^*th*^ element. Once the above superposition is computed, the corresponding time series can be obtained via its inverse transform. The transformations between the time and frequency domains are achieved using Intel Math Kernel Library (MKL) functions. A detailed description of this method, including validation against experimental measurements and 3D direct numerical simulations have been previously described (Seo et al., 2017).

## Results

### Simulation Setup

**FIGURE 6:**
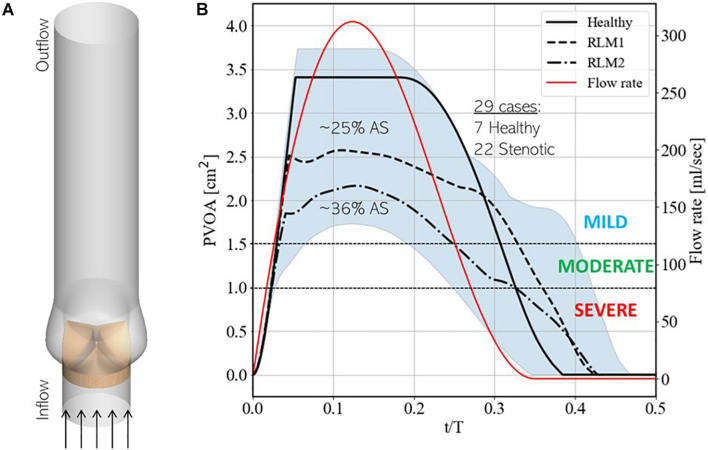
**(A)** Schematic showing simulation setup and **(B)** instantaneous upper and lower bounds on projected valve open area (PVOA) in the training dataset, indicated by the blue shaded region, with three baseline simulations, indicated using solid, dashed and dashed-dot black lines. These simulations correspond to cases 7, 13, and 20, respectively, from Supplementary Table 1 in Appendix A. Additionally, the secondary *y*-axis describes the instantaneous flow-rate profile used to drive simulations.

Our simulation dataset consists of 29 simulations with 7 healthy and 22 stenotic valves. Aortic stenosis is induced by increasing the stiffness parameter κ in one or more valve leaflet over its baseline value such that area stenosis is at most mild. Details of individual simulations, including leaflet mobility and area stenosis, are listed in Supplementary Appendix A. Simulations are driven by a plug velocity profile at the LVOT (Figure 6A) and a flow-rate profile (Figure 6B) based on echocardiographic measurements from patients with aortic stenosis (Barletta et al., 2018). Two stroke volumes (60 and 72 ml) are tested, and heart-rate is assumed to be 60 bpm, such that the corresponding cardiac outputs are 3.6 and 4.32 lpm. The outflow boundary uses a zero-gradient based boundary condition to ensure global mass-conservation. A zero-gradient pressure boundary condition is prescribed at the inflow boundary, while pressure at any point in the aorta is measured relative to that at the outflow boundary.

Figure 6B also shows the upper and lower bounds on the time-varying projected valve open area (PVOA) from the complete dataset and highlights three representative cases of different types of valve conditions tested in the dataset. Of these, the first describes a ‘Healthy’ case, in which all three leaflets exhibit full range-of-motion (ROM), and the other two describe stenotic cases, in which one or more leaflet experience reduced leaflet motion (RLM). Thus, the first stenotic case is labeled ‘RLM1’ with one leaflet stiffened compared to the other two and the second is labeled ‘RLM2’ with two leaflets stiffened compared to the third. Compared to the Healthy case, the RLM1 and RLM2 cases represent approximately 25 and 36% area stenosis, and the three cases correspond to simulation numbers 7, 13, and 20, respectively, in Supplementary Table 1 of Appendix A. The PVOA plots are examined in the context of the American Heart Association’s guidelines for determining AS severity (Nishimura et al., 2014), which indicates both RLM cases as being classified as mildly stenotic (peak PVOA ≥ 1.5 cm^2^). In fact, all 29 simulated cases are healthy or mildly stenotic, which is important since early detection of AS onset is the goal of this investigation. In the following section, differences in blood flow patterns and the consequent acoustic response recorded at a monitoring point are described.

### Hemodynamics of Healthy and Stenotic TAVs

**FIGURE 7:**
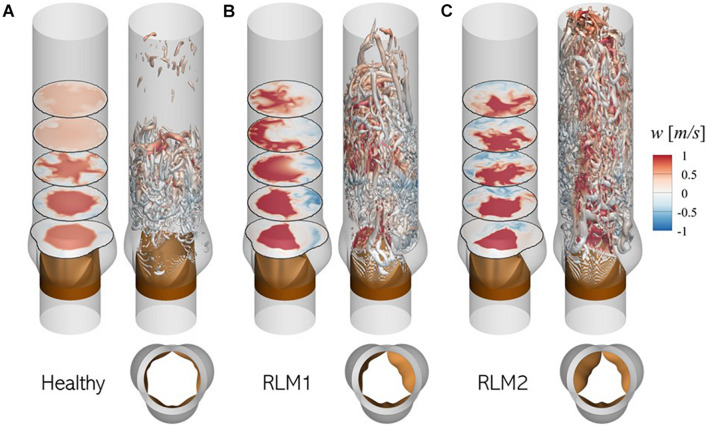
Illustration of flow physics and aortic jet dynamics in **(A)** Healthy, **(B)** RLM1, and **(C)** RLM2 cases. In each case, the left panel shows contours of axial velocity in five axial planes: 1.5, 3.0, 4.5, 6.0, and 7.5 cm from the aortic annulus and the right panel shows iso-surfaces of Q-criterion (Q = 10^4^ m/s^2^) colored by axial velocity. Red colors indicate antegrade flow and blue colors indicate retrograde flow. Large regions of forward flow in a section plane can be used to identify aortic jet shape in that plane.

Aortic valve hemodynamics play an integral role in assessing valve performance and diagnosing valve health: transvalvular gradient, peak jet velocity and their derivatives are commonly used to infer aortic stenosis severity (Nishimura et al., 2014). Changes in downstream flow features, such as jet trajectory, flow kinetic energy and turbulence cause changes in local pressure fluctuations on the aorta lumen boundary, which ultimately defines the acoustic response from the aorta. Therefore, it is important to understand post-valvular hemodynamic differences resulting from healthy and stenotic valves. For the three baseline simulations described above, flow features during early deceleration (∼*t*/*T* = 0.20) are illustrated in Figure 7.

In the healthy case the aortic jet, visualized as contours of forward flow (red) in axial planes, appears circular symmetric due to equal mobility on all three leaflets. The jet diffuses as it propagates downstream and reattaches with the aorta lumen boundary ∼ 6.0 cm from the aortic annulus. Upstream from this reattachment point, the jet is surrounded by an annulus of retrograde flow (blue) where turbulent vortex structures are formed. These turbulent vortices are formed in the shear layer between the aortic jet and the surrounding flow, due to the adverse pressure gradient experienced by the flow during deceleration.

In stenotic cases (RLM1 and RLM2), the aortic jet tilts away from leaflets exhibiting RLM and shows significant shape distortion as it impacts the aorta lumen boundary. Consequently, jet reattachment point moves further away from the valve. At the same time, regions of retrograde (or recirculating) flow develop downstream from the RLM leaflets. Due to a smaller effective orifice area, the jet velocity is larger in the stenotic cases as opposed to the healthy case, as evidenced by larger downstream travel of the jet in the same interval. The increased jet velocity, tilting and distortion contribute to flow separation, in addition to the adverse pressure gradient, resulting in significantly more turbulent vortex structures. These differences ultimately influence the sound generated by the cardiovascular unit and that recorded by a sensor. A more detailed description of transvalvular hemodynamics in healthy and stenotic valves, including peak jet velocities, jet asymmetry and surface loading on lumen boundary, can be found in our recent study (Bailoor et al., 2021a).

### Acoustic Response Recorded on the Thorax

The typical acoustic response from a healthy aortic valve is a distinct “click” sound at the end of systole, indicating aortic (and pulmonary) valve closure and is referred to as the second (or “S2”) sound. A stenotic aortic valve, on the other hand, results in a harsh mid-systolic murmur (Grimard et al., 2016), heard as a “whooshing” sound, which is loudest over the second right intercostal space and radiates to the carotid arteries. Due to the high-pressure gradient across stenotic AVs, this murmur is usually higher in pitch compared to other cardiac murmurs (Grimard et al., 2016). The stenotic valve murmur may be accompanied by ejection clicks, 40–60 ms following the first heart sound (or “S1”), at maximal valve opening (Jacobs, 1990) and even diminished or absent second sound (Grimard et al., 2016). We analyze acoustic signals from our simulations in the context of these features.

**FIGURE 8:**
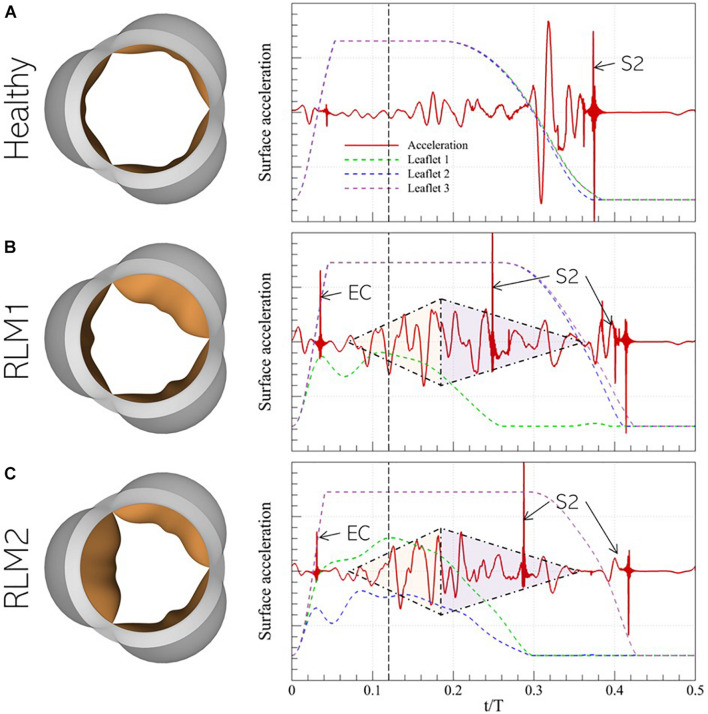
(left) Peak systolic valve configuration and (right) systolic surface acceleration measurement at the monitor point in the representative **(A)** Healthy, **(B)** RLM1, and **(C)** RLM2 cases. The colored dashed lines show instantaneous displacement of the three leaflets in each case and the vertical dashed black line denotes the instant of peak systole. Key instances of systolic heart sounds are also indicated: EC- ejection click, S2- “dub”/second heart sound, shaded triangles are used to mark the crescendo/decrescendo of the stenotic valve murmur.

The normalized surface acceleration recorded at the monitor point shown in Figure 5B for the three cases described in section 3.2 are illustrated in Figure 8 (right). The corresponding valve sounds can be heard over 5 cardiac cycles using the video files provided in the Supplementary Data. Due to the strong coupling between instantaneous valve configuration and valve sound, it is useful to examine these signals in relation to the time-varying leaflet displacement, indicated using colored dashed lines. A vertical black, dashed line is also plotted at *t*/*T* = 0.12 to indicate peak systole. This helps separate the phases of systolic ejection and explain surface acceleration in the context of these phases.

In the Healthy case, the signal is observed to be quiet during acceleration when flow is largely laminar. During deceleration, the signal amplitude grows, starting right before the onset of leaflet closure (∼*t*/*T* = 0.20) and continuing until ∼*t*/*T* = 0.30. At this point, the leaflets are halfway closed, and the acoustic response shows strong oscillations. Finally, when the leaflets close ∼*t*/*T* = 0.38, large amplitude spikes are observed which correspond to the second (S2) sound. In both stenotic cases, several common features are observed: an almost linear increase in the amplitude (or crescendo) of the signal with time, starting around mid-acceleration (∼*t*/*T* = 0.06) through early deceleration (∼*t*/*T* = 0.18), followed by a period of decreasing amplitude (or decrescendo) starting around *t*/*T* = 0.18 and extending through the end of systole. Thus, this time interval shows the presence of the characteristic diamond-shaped murmur of stenotic valves, indicated by shaded triangles. Moreover, the first click sound occurs early in acceleration (∼*t*/*T* = 0.06). Compared to the stenotic cases, this sound is significantly less pronounced and is in fact inaudible in the Healthy case. Its occurrence, following the onset of systolic ejection (or end of diastole/S1), is consistent with the timing of ejection clicks in stenotic valves. However, contrary to the belief that ejection clicks occur at maximum valve opening (Jacobs, 1990), we observe they coincide with the instant the stiffest leaflet in the valve first changes direction of travel (opening-closure). Another interesting observation is the splitting of the S2 sound, occurring due to the early closure of the stiffer leaflets and the delayed closure of the healthy leaflets. Ordinarily, S2-splitting is attributed to asynchronous closure of aortic and pulmonic valves, but our results show that asynchrony in leaflet closure in the same valve can also cause multiple sounds. Another notable feature of the stenotic valves is the relatively weak acoustic response immediately preceding the final S2 sound. Due to a differential in leaflet stiffness, closure of healthy leaflets shows a significant time-lag compared to that of stiffer leaflets. For instance, in the RLM1 case, leaflets 2 and 3 close at ∼*t*/*T* = 0.42, which is 160 ms behind the closure time for leaflet 1, whereas in the RLM2 case, leaflet 3 closes 120 ms after leaflets 1 and 2, at ∼*t*/*T* = 0.42. Contrast this with the healthy case, in which all leaflets close together at ∼*t*/*T* = 0.38. Systolic ejection takes place when *t*/*T* ∈ [0.00−0.35], and the strong acoustic response in the Healthy case occurs when all leaflets are rapidly closing (∼*t*/*T* = 0.30) against forward flow. This creates large pressure fluctuations in the valve vicinity, and consequently, the stronger response. In the stenotic cases, the stiffer leaflets close during mid-deceleration ∼(*t*/*T* ∈ [0.26−0.30]), leaving the Healthy leaflets open to admit the remaining forward flow. As a result, the healthy leaflets stay open longer, past the systolic ejection period. The halfway closure instants for the Healthy leaflets in the RLM1 and RLM2 cases are at ∼*t*/*T* = 0.36 and ∼*t*/*T* = 0.38, respectively, which are both after the end of forward flow, meaning the leaflets are closing in the absence of opposing forward flow. This delay in closure, beyond the systolic ejection period results in a diminished/absent acoustic response following systolic murmur.

It is evident from Figures 7, 8 that healthy and stenotic aortic valves have distinct hemoacoustic features which are intimately tied to valve function. It should therefore be possible to leverage supervised learning techniques to “understand” these differences and accurately predict valve status (Healthy/Stenotic) for new instances by analyzing its associated acoustic response. We accomplish this using linear discriminant analysis (Kutz, 2013) for which a detailed description of the process is described in Supplementary Appendix B.

### Detecting Anomalous Valve Function

**FIGURE 9:**
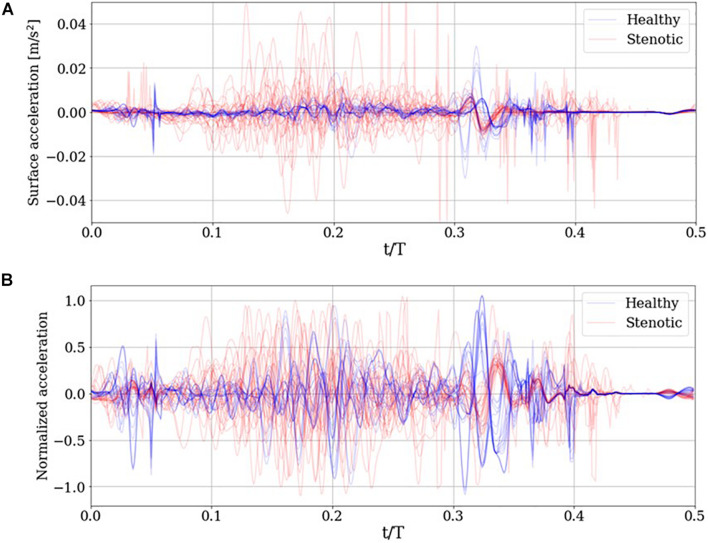
**(A)** Simulated surface acceleration recorded on the thorax and the **(B)** corresponding centered and normalized acceleration for the 29 simulations and 15 synthesized healthy signals.

Once surface acceleration signals are computed at the monitor location for all simulations, they are processed to train a classification algorithm. The first step in this processing in dataset balancing to avoid biasing the classifier in favor of the majority (in this case, “Stenotic”) class. The imbalance in the dataset arises from the fact that there is only one “Healthy” configuration, in which all leaflets exhibit full range-of-motion. In contrast, any deviation from this configuration would be an example of stenosis. Thus, many more combinations of leaflet mobility can be simulated to populate the stenotic class, resulting in a naturally unbalanced dataset. To overcome this issue, we synthesize 15 additional healthy class signals as combinations of the 7 healthy signals available in the dataset, using Synthetic Minority Oversampling TEchnique (SMOTE). The technique and its application to our dataset are described in detail in Supplementary Appendix C. The resulting balanced dataset of surface acceleration signals are shown in Figure 9A.

The surface acceleration *a_i* for simulation ‘*i*,’ recorded at thoracic location $\vec{x}$_*m*_, has ‘*N*’ time samples. Using a sampling frequency of 10 kHz over a period of 0.5 s results in *N* = 5000. Each signal is normalized to unit magnitude then centered about the ensemble average $\bar{a}$ of the normalized dataset as follows:


(13) $$a_{i}\,\left(\vec{x},t\right)=\left[a_{i}\,\left(\vec{x},t_{1}\right),a_{i}\,\left(\vec{x},t_{2}\right),\cdots ,a_{i}\,\left(\vec{x},t_{N}\right)\right]$$

$$a_{i}^{*}\,\left(\vec{x},t\right)=\frac{a_{i}\,\left(\vec{x},t\right)}{{||a_{i}\,\left(\vec{x},t\right)||}_{\infty }},\bar{a}\,\left(\vec{x},t\right)$$

(14) $$\bar{a}\,\left(\vec{x},t\right)=\frac{1}{M}\,\sum_{i=1}^{M}a_{i}^{*}\,\left(\vec{x},t\right)$$

(15) $$a_{i}^{\prime }\,\left(\vec{x},t\right)=a_{i}^{*}\,\left(\vec{x},t\right)-\bar{a}\,\left(\vec{x},t\right)$$

In the above equations, *M* indicates the dataset size (*M = 44*). The resulting normalized and centered signals $a_{i}^{\prime }\,\left(\vec{x},t\right)$ are illustrated in Figure 9B. Next, we reduce the dimensionality of the dataset using principal component analysis (PCA). This serves two purposes: first, by reducing the dimensionality of the dataset, we can instead work with a significantly compressed representation of the dataset which reduces computational costs associated with classification. The second advantage, specific to PCA, is that dimensionality reduction is done based on explained variance. This means that one can choose a reduced-rank representation of the dataset which accounts for a desired fraction of the total variance in the dataset. The individual acceleration signals are arranged in a data matrix A, then its reduced-rank representation is obtained via its singular value decomposition (SVD), as shown in equation (16):


(16) $$A={\left[\begin{array}{c} \begin{array}{c} a_{1}^{\prime }\,\left(\vec{x},t\right)\\ a_{2}^{\prime }\,\left(\vec{x},t\right) \end{array}\\ \begin{array}{c} \vdots \\ a_{M}^{\prime }\,\left(\vec{x},t\right) \end{array} \end{array}\right]}_{\left(M\times N\right)},\begin{array}{c} A=U\,\Sigma \,V^{T}\\ V_{P}=\left[\begin{array}{cc} \begin{array}{cc} \vec{v} & \vec{v} \end{array} & \begin{array}{cc} \cdots & \vec{v} \end{array} \end{array}\right]\\ A_{P}=U\,\Sigma_{P}\,V^{T} \end{array}$$

In the above equation, U and V are orthonormal matrices of dimensions (*M*×*M*)*and*(*N*×*N*), respectively. The column vectors of U and V are referred to as left and right singular vectors, respectively. Σ is an (*M*×*N*) diagonal matrix containing min(*M*,*N*) singular values in descending order, such that ∑_11_ > ∑_22_ > ∑_33_… Individual singular values are proportional to the amount of variance in *A* (assuming *A* is centered) explained along the mode represented by the corresponding columns of U and V. A low-rank reconstruction of *A* can be calculated by blanking all but the first *P* diagonal entries in *Σ* such that *A*_*P*_ = *U*Σ_*P*_*V^T^* is the *P*-mode reconstruction of *A*, and which accounts for the total variance explained by the first *P* singular modes. Next, we project individual acceleration measurements on the first *P* right singular vectors to get a feature vector of size *P*, as shown in equation (17), which is later used for classification:


(17) $$\Omega_{i}=\vec{a}\cdot V_{P}=\left[\begin{array}{cc} \begin{array}{cc} \omega_{1} & \omega_{2} \end{array} & \begin{array}{cc} \cdots & \omega_{P} \end{array} \end{array}\right]$$

In this manner we reduce the dimensionality of the dataset from (*M*×*N*) to (*M*×*P*) with *P*≪*N*. Once a classifier is chosen and the SVD of A is computed, a choice of *P* must be made to compute the feature matrix **Ω**, and to tune the classifier for optimal performance. One way to choose P would be by deciding a minimum desired fraction of the total variance to be explained by the reduced dataset, then using the fewest P modes, which cumulatively account for at least the desired fraction. The downside of this approach is that the minimum desired variance is likely arbitrary, which if too small would not contain enough information from the development set, and if too large would risk overfitting to the development set. In either case, the classifier’s predictive accuracy would deteriorate. We instead determine *P* via cross-validation, in which a large fraction of the development set is used for training the classification algorithm, and the remaining, smaller subset is reserved for validating the algorithm. The optimal value of *P* was determined to be 19, as elaborated in Supplementary Appendix D. The validation set is then incorporated into the development set and the LDA classifier is trained with the complete dataset. To account for the additional variance introduced by the validation set, the number of PCA modes used for training is increased to 20.

**FIGURE 10:**
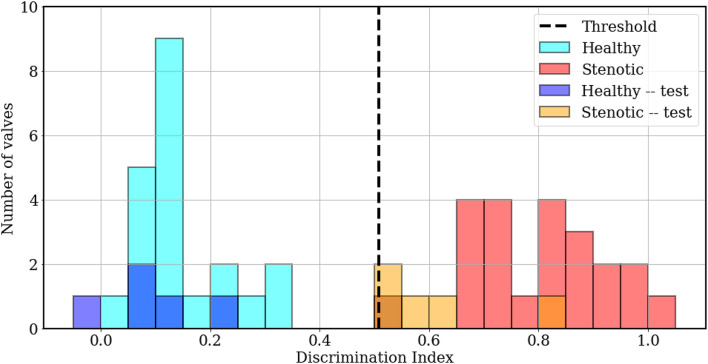
Histogram plots showing classification results, computed as LDA projection values of individual simulations from the training (cyan, red) and test (blue, orange) sets, relative to the threshold value (dashed black line).

Next, we wish to assess whether the developed classifier can prospectively detect the presence of abnormal valve function in new instances. To do this, we generated a test set of 5 mildly stenotic valves and additionally synthesized 5 healthy signals from the development set. Care was taken to ensure that the simulation (or interpolation) parameters for these signals did not coincide with any signals from the development set and a detailed description of this dataset is provided in Supplementary Appendix A. Classification results from both datasets are illustrated in Figure 10. The discrimination boundary, denoted by the dashed black line, is computed as the mean value of the largest LDA projection from the healthy class and the smallest projection from the stenotic class, excluding any overlapping projections. It is observed this simple criterion of determining a discrimination threshold is capable of retrospective as well as prospective detection of valve failure: all valves in the training set were accurately classified, while one false negative was incurred in the test set. There is some uncertainty in predictions where the LDA projections take values ∈ [0.50−0.55]. It is observed the one false negative was observed for case 1 from the test set, which represents ∼20% area stenosis (AOA = 2.97 cm^2^) and corresponds to less than 5 mmHg peak systolic gradient. It should be noted that such stenosis is considered subclinical and, assuming normal ventricular function, the patient would likely not experience heart failure symptoms. To summarize, we were able to achieve 100% accuracy for retrospective detection (training) and 90% accuracy for prospective prediction (testing) of abnormal valve function using PCA-based dimensionality reduction and LDA.

In section 4, we discuss the role of important PCA modes and relevant temporal features of the recorded signals in the classification process.

## Discussion

**FIGURE 11:**
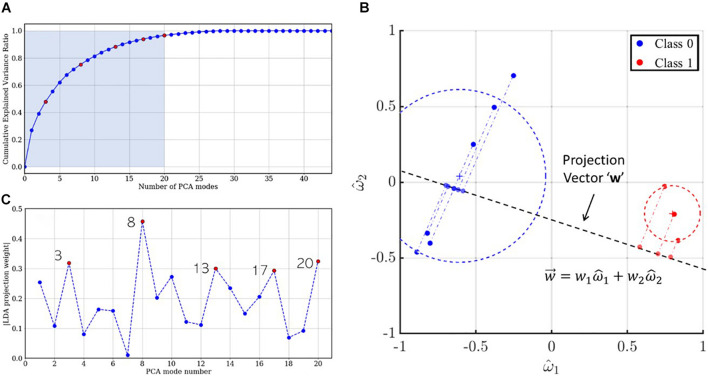
**(A)** Scree plot indicating the cumulative explained variance ratio for the singular value spectrum. The five most important modes for LDA projection are colored using red circles. **(B)** Two-dimensional illustration of LDA projection for a sample dataset using the optimal projection vector $\vec{\text{w}}$. **(C)** LDA projection weights for the first 20 PCA modes and the five most important LDA modes colored in red.

In a previous section, we mentioned that PCA facilitates variance-based dimensionality reduction. An effective reduced-order model for the dataset assumes that the underlying dynamics have prominent low-rank features. In PCA, a reduced-order model is reconstructed as a linear combination of orthonormal modes, which are arranged in decreasing order of explained variance. Thus, the first mode is expected to contain information about the dominant, largest-scale features, while higher modes progressively account for sparser, smaller scale features. This is evident from the cumulative explained variance ratio of singular value spectrum illustrated in Figure 11A. Recall that the development set has 44 (= *M*) PCA modes of which the first 20 (shaded), accounting for more than 95% information, were used for training the LDA classifier. While these modes are arranged in descending order of explained variance, the relative importance of each PCA mode may follow a different order for classification. This order is given by the absolute value of the coefficients associated with the corresponding bases for the LDA projection vector $\vec{w}$, hereafter referred to as “LDA projection weights.” For example, consider the sample dataset in two-dimensional feature space, shown in Figure 11B: the blue and red circles represent mappings from the “0” and “1” class, respectively, the LDA projection vector (dashed line) is written as $\vec{w}=w_{1}\,{\hat{\omega }}_{1}+w_{2}\,{\hat{\omega }}_{2}$. The projections of the dataset on this vector are also shown using translucent dashed-dot lines. It is evident that ||*w*_1_|| > ||*w*_2_|| and projections of the dataset on the ${\hat{\omega }}_{1}$ basis would yield better class separation than along ${\hat{\omega }}_{2}$. Thus, for the 20-dimensional optimal projection vector for the training set, the mode-wise LDA projection weights are plotted in Figure 11C. It is evident from the non-monotonic plot that the relative importance of the PCA modes does not follow the same order as the singular values. The five most important PCA modes are marked using red circles.

**FIGURE 12:**
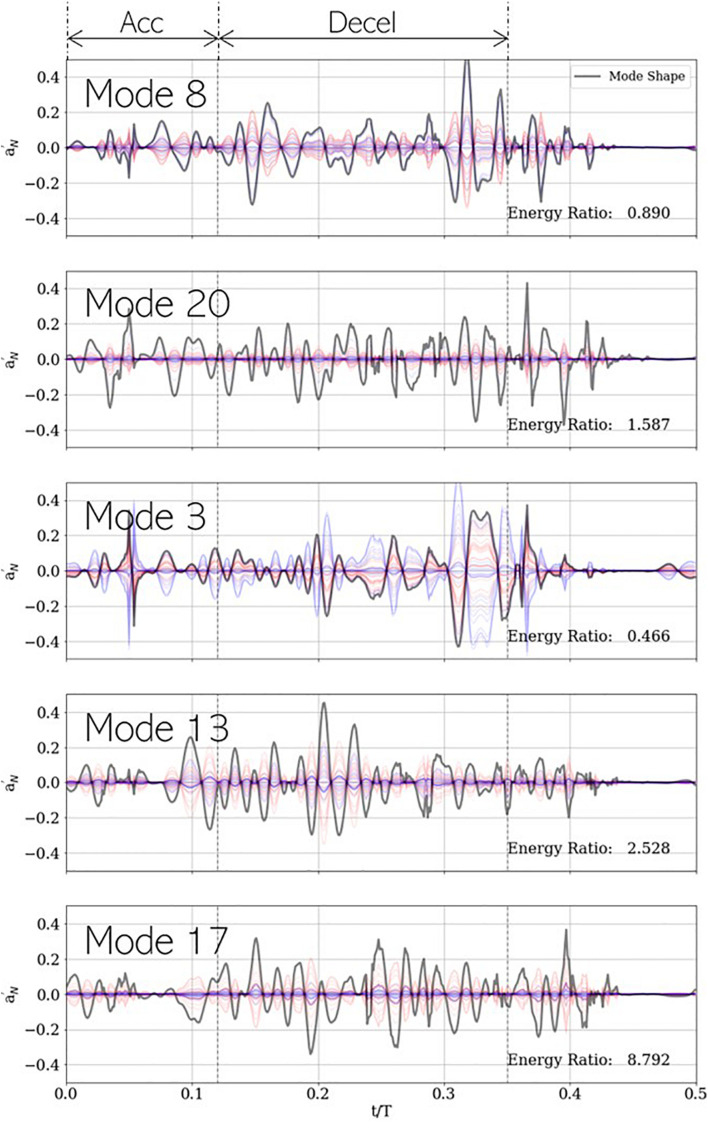
Five most important PCA modes for LDA-based classification, shown with each mode shape (gray) and its corresponding contribution to the development dataset. Blue and red plots represent healthy and stenotic valves, respectively. Vertical dashed black lines at **t**/**T**=**0.12**and**0.35** indicate the ends of acceleration and deceleration phases of systolic ejection, respectively. For each mode, energy ratio is listed in the bottom-right corner of the corresponding panel.


The mode shape for a specific PCA mode is given by the corresponding column vector of the right singular matrix ***V***, shown using gray lines in Figure 12. Likewise, the contribution of mode ‘*j*’ to a given measurement ‘*i*’ is given by ${\vec{a}}_{i,j}^{\prime }=\Sigma_{j\,j}\,U_{i\,j}\,{\vec{v}}_{j}$, which is a scalar multiple of the *j*^*th*^ right singular vector. This is evidenced by the corresponding single-mode reconstructions of individual case signals. For accurate classification using any given mode ‘*j*,’ the coefficients $\Sigma_{j\,j}\,{\vec{u}}_{j}$ must show class wise differences in (a) sign or (b) magnitude. For example, in mode 3, healthy (blue) and stenotic (red) signals appear to be separated by sign, while in modes 13, 17, and 20, the classes appear divided by intensity. To quantify this, we compute an energy ratio (*ER*_*j*_) for mode ‘*j*,’ defined as the ratio of the mean squared vector-norm of the *j-*mode reconstruction of the stenotic class to that of the healthy class, as shown in equation (18):

(18) $$E\,R_{j}=\frac{\left[\sum_{i\in N_{S}}{||{\vec{a}}_{i,j}^{\prime }||}^{2}\right]/N_{s}}{\left[\sum_{i\in N_{S}}{||{\vec{a}}_{i,j}^{\prime }||}^{2}\right]/N_{H}}$$

where *N_S* and *N_H* represent number of valves in the healthy and stenotic classes, respectively. This metric describes the relative importance of a specific mode, on average, to each class of valves for classification. It is observed ER < 1 for modes 3 (0.466) and 8 (0.890) and ER > 1 for modes 13 (1.587), 17 (2.528), and 20 (8.792). It is unsurprising the lower-rank (3 and 8) modes are on average more energetic in healthy valves, and the higher modes (13, 17, and 20) are more energetic in stenotic valves, but an understanding of the important temporal features of each mode can help gain insight into the role each mode plays in classification.

Modes 3 and 8 show highest peaks late in deceleration (*t*/*T* ∈ [0.30−0.35]), around the time when valve leaflets close. This is aligned with our expectation that lower-rank modes would describe dynamics associated with large-scale fluctuations in the system dynamics. Moreover, it was observed that healthy valves show a strong acoustic response in this interval due to simultaneous closure of all leaflets and that this response is diminished in stenotic valves. This is reflected in the low values of ER for these modes. Mode 3 also shows a large spike around mid-acceleration (*t*/*T* ∈ [0.04−0.06]), which corresponds to the interval of ejection clicks in stenotic valves. Recall that we are analyzing mode shapes of the centered dataset and the ensemble average contains an imprint of this ejection click, which is why it is also seen in the mode 3 reconstruction of healthy signals. On the other hand, modes 13, 17, and 20 show prominent peaks all through deceleration and are more energetic for stenotic valves. This interval coincides with the generation of systolic murmurs, which are typically absent in healthy valves as evidenced by high ER values, exceeding factors of 8.

To summarize, the LDA-based classifier can discriminate healthy from stenotic valves based on differences in the following temporal features of the associated acoustic signals, recorded by a sensor on the patient’s thoracic surface:

(a) Low-rank (or large-scale) features corresponding to valve closure sound, occurring toward the end of systolic ejection.
(b) Presence of systolic ejection clicks occurring early in systole (mid-acceleration).
(c) Sparser, high-rank (or high frequency) features corresponding to stenotic valve murmurs.

Differences in these features are clinically observed, as described in section “Acoustic response recorded on the thorax”, and are well-replicated by our CHA solver. This demonstrates that a data-driven methodology can be potentially used to lay the groundwork for an inexpensive and non-invasive valve monitoring technology, capable of early and accurate detection of the onset of valve failure. Moreover, we also show how computational modeling can play a crucial role in the development of such technology.

## Concluding Remarks

This study lays the groundwork for a practical and accessible auscultation-based longitudinal monitoring modality to screen for aortic valve systolic murmurs, and ultimately, mild or subclinical stenosis secondary to early leaflet thrombosis. This is achieved through data-driven analysis of computational modeling of the hemoacoustic phenomena in and surrounding the human ascending aorta. The complex blood flow and aortic valve interactions are simulated using a finite-difference based flow solver, a simple reduced order valve model and a sharp interface immersed boundary method. The surface pressure on the aorta lumen boundary is treated as a source for the generation and propagation of elastic waves through a homogenous medium which serves as the surrounding tissue. Wave propagation is simulated in the frequency domain using the free space Green’s function. We demonstrated that with healthy valves, the prominent acoustic sound occurs near the end of systolic ejection due to the simultaneous closure of the three valve leaflets (called “S2”). On the other hand, asymmetric reduced leaflet motion in stenotic valves results in aortic jet tilting and impingement on the lumen boundary, increased retrograde flow and turbulent vortex structures. These conditions result in the generation of systolic murmur which follow “crescendo-decrescendo” patterns and a diminished closure sound. When pathologically stiff leaflets first change direction of travel, early in systole, “ejection clicks” can be heard from stenotic valves. All these clinically observed features are well replicated from our models. Additionally, our simulations also show a splitting of “S2” sound due to asynchronous closure of stenotic valve leaflets, leading to multiple closure clicks.

We used acoustic data from 29 such direct numerical simulations of healthy and mildly stenotic valves and 15 additional synthesized healthy signals to train a linear discriminant analysis-based classification model. Dimensionality reduction using principal component analysis was performed prior to training, such that the actual development set is substantially smaller than the collection of raw signals while preserving most of the contained information. The trained classifier was tested on a new dataset with 5 stenotic valves and 5 synthesized healthy signals and was shown to have 100% retrospective and 90% prospective accuracy in prediction. Finally, we showed that the 5 most important principal component modes for classification identified healthy valves as having a prominent closure sound signature and an absence of systolic murmur. Whereas, for stenotic valves the PCA modes primarily contained information regarding systolic murmur. Thus, simple dimensionality reduction techniques retain clinically important features of healthy and stenotic aortic valve sounds, which can be analyzed to detect mild, and even subclinical aortic stenosis, secondary to early leaflet thrombosis.

This investigation, however, is preliminary and has some limitations which must be addressed before clinical translation. For instance, the parameter space over which the classification algorithm is trained is relatively small, and includes variations in leaflet mobility, flow profiles and stroke volume (see Supplementary Table 1). However, the generation of turbulent pressure fluctuations on the lumen boundary and consequently, heart murmurs, depends on several other factors, such as secondary flow features like flow helicity due to ascending aorta curvature, LVOT ellipticity and heart rate. To account for these variables, we are adapting this analysis to flow simulations in realistic, patient-specific aorta anatomies, obtained from cardiac CT and subject to a variety of physiological flow conditions and ventricular functions. Our model would also benefit from accounting for systolic dilation of the aorta due to vascular compliance and the consequent acoustic response. Moreover, the acoustic response recorded on the thorax also depends on the patient’s body habitus, variations in which can be considered by changing the monitoring point location. Clinical PCG signals are often corrupted with noise and breathing sounds due to inspiration/expiration and these characteristics can interfere with the algorithm’s performance. We plan on including realistic levels of signal corruption and appropriate denoising strategies in our future work to address this issue.

Nevertheless, the methods discussed herein can lead to a new paradigm of patient-friendly, inexpensive, non-invasive, and non-toxic modality for bioprosthetic valve monitoring upon further development. The safety and ease of an auscultation-based method, together with improved pattern recognition accuracy using machine learning makes at home monitoring possible without the need for trained personnel or physicians. While the motivation for this work is rooted in issues faced by transcatheter valves, the applicability of the developed methods extends to surgical and even native valves.

## Data Availability Statement

The original contributions presented in the study are included in the article/Supplementary Material, further inquiries can be directed to the corresponding author/s.

## Author Contributions

SB designed the study, contributed analysis tools, collected the data, analyzed the results, and wrote the manuscript. J-HS designed the study, contributed analysis tools, analyzed the results, and wrote the manuscript. SS analyzed the results. RM conceived the study, designed the study, and analyzed the results. All authors contributed to the article and approved the submitted version.

## Conflict of Interest

The authors declare that the research was conducted in the absence of any commercial or financial relationships that could be construed as a potential conflict of interest.

## Publisher’s Note

All claims expressed in this article are solely those of the authors and do not necessarily represent those of their affiliated organizations, or those of the publisher, the editors and the reviewers. Any product that may be evaluated in this article, or claim that may be made by its manufacturer, is not guaranteed or endorsed by the publisher.
