# Supplementary material for: Detecting Aortic Valve Anomaly From Induced Murmurs: Insights From Computational Hemodynamic Models
Source: Front Physiol. 2021 Oct 6;12:734224. doi: 10.3389/fphys.2021.734224 (PMC8526559; doi:10.3389/fphys.2021.734224)
Supplement: Supplementary file 4 [file Data_Sheet_1.PDF]

Supplementary Material

1 Appendix A: Development and test sets:

The development and test sets are shown in Table S**1** and Table S**2**, respectively, with parameter values listed for each simulation. As observed in the tables, we observed several valve and flow parameters, such as valve area (through leaflet stiffness), leaflet mapping function, stroke volume and flow profile. The peak anatomic orifice area (AOA) in every case is larger than 1.5 cm^2, ensuring stenosis severity is at worst mild.

2 Appendix B: Linear Discriminant Analysis:

We use linear discriminant analysis (LDA), as described by Kutz (Kutz 2013), as the classification algorithm for detecting anomalous valve function. LDA is a dimensionality reduction technique which seeks to find an optimal projection vector '$\vec{w}$', such that when the original binary class dataset is projected on to this vector (thus reducing its dimensionality to 1), class separability is maximized. This can be visualized using a sample two-dimensional dataset, illustrated in Figure S1 (a). The blue dots represent the distribution of Healthy valves while the red dots represent that of the Stenotic valves. Additionally, three projection vectors, along with the corresponding projections of the dataset, are shown in Figure S1 (b), (c) and (d), as examples of "bad", "good" and "optimal" class separability, respectively. Through these images, it is easy to intuitively understand what optimal projection requires: Maximal class separation and, simultaneously, tight within-class clustering. Mathematically, class separability is quantified using the Fisher Discriminant Ratio ($J(\vec{w})$), defined as follows:

$$J(\vec{w}) = \frac{(\mu_H - \mu_S)(\mu_H - \mu_S)^T}{\sum_{i \in H}(\tilde{\Omega}_i - \mu_H)(\tilde{\Omega}_i - \mu_H)^T + \sum_{i \in S}(\tilde{\Omega}_i - \mu_S)(\tilde{\Omega}_i - \mu_S)^T} \tag{S1}$$

In equation (S1), μ_H and μ_S denote the mean value of the Healthy and Stenotic classes when the given dataset is projected on to the vector $\vec{w}$. $\tilde{\Omega}_i$ is the projection of the ith class from the original dataset on to $\vec{w}$. Thus, the numerator can be understood as the squared distance between the mean values of the two classes under projection on to $\vec{w}$, or more generally, the between-class scatter (S_B). This quantity represents how far apart the class means move under LDA projection. On the other hand, the denominator can be understood as the sum of within-class scatter (S_W) for the two classes. Each term in the denominator is proportional to the variance of the corresponding class about its mean, under LDA projection.

Thus, finding optimal class separation is equivalent to finding a vector $\vec{w}$ which maximizes this ratio, and is determined as the solution to the following generalized eigenvalue problem:

$$\boldsymbol{S}_B\vec{w} = \lambda \boldsymbol{S}_w\vec{w} \tag{S2}$$

where $\boldsymbol{S}_B$ and $\boldsymbol{S}_W$ are matrices corresponding to the between-class and within-class scatter for the original dataset. The optimal $\vec{w}$ is given by the eigenvector corresponding to the largest eigenvalue λ_{max}.

3 Appendix C: Synthetic Minority Oversampling Technique (SMOTE):

This technique is commonly employed in statistical analysis when training a machine learning algorithm on an unbalanced dataset, i.e. objects in one class outnumber those in the other, can risk introducing bias in the algorithm's predictions. SMOTE helps to oversample the minority class and generate sufficiently many instances of the class in a given feature space, such that the dataset is then balanced. The methodology is illustrated for a sample two-dimensional dataset in Figure S2 (a).

To generate additional samples, a random instance from the minority class is selected, hereafter referred to as "central instance" (circled), and its 'k'-nearest neighbors determined (blue circles within larger blue shaded circle). Then, for each neighbor, a minority class instance is synthesized as a random convex combination of the central instance and the neighbor, shown using green stars. This process is repeated, each time with a new central instance, until the datasets are balanced.

In our case, since dataset balancing is performed before principal components are calculated, each time sample is treated as an independent feature. For a central instance 'i' and a generic neighbor 'j', a distance metric (equation (S3)), calculated as the squared norm of the difference between their measured surface acceleration signals, is tested to determine its 'k' nearest neighbors. The resulting balanced dataset is illustrated in Figure S2 (b).

$$d_{i,j} = \int_0^T \left(\vec{a}_i - \vec{a}_j\right)^2 dt \qquad \text{(S3)}$$

4 Appendix D: Parameter tuning via Cross-Validation:

The primary model parameter for the PCA-based linear discriminant analysis classifier is the number of PCA modes, P. As described in section 3.4, this may be achieved by arbitrarily fixing the amount of explained variance in the reduced dataset. Another way, which also gives an a priori estimation of the predictive accuracy of the classifier is via K-fold cross-validation. This method is outlined as follows:

1. Randomly shuffle the development dataset.
2. Split the dataset into 'K' groups. The first two steps are done using the Python Scikit-Learn's KFold model selection library. One group is set aside for validation (or testing), while the remaining K-1 are used for model training.
3. Train the LDA classifier using the K-1 groups using different possible values of P and evaluate its performance on the remaining validation group. Classification error is assessed as: $CE = (FP + FN)/(TP + TN + FP + FN)$, where TP, FP, FP, and FN represent the number of true positives, true negatives, false positives, and false negatives incurred on a given training or validation set.
4. This process is repeated using every group for validation and the remaining K-1 for training.

5. For a given P, the average classification error on a given dataset is calculated as the average error over all tested combinations of simulations used for that dataset.

The trends of mean classification error on the training and validation sets as the number of PCA modes 'P' is varied are illustrated in Figure S3. The training error generally shows a monotonic decrease as 'P' is increased, and eventually decreases to zero, indicating the classifier contains all the information required for classification on the training set. On the other hand, the mean validation error first decreases between $6 < P < 14$, then increases for $P > 19$, which coincides with the region where the mean training error is zero. This indicates the classifier is now overfit to the training set and loses generalization accuracy on new instances. Thus, optimal classification occurs when the mean validation error is minimal, at $P = 19$.

Figures:

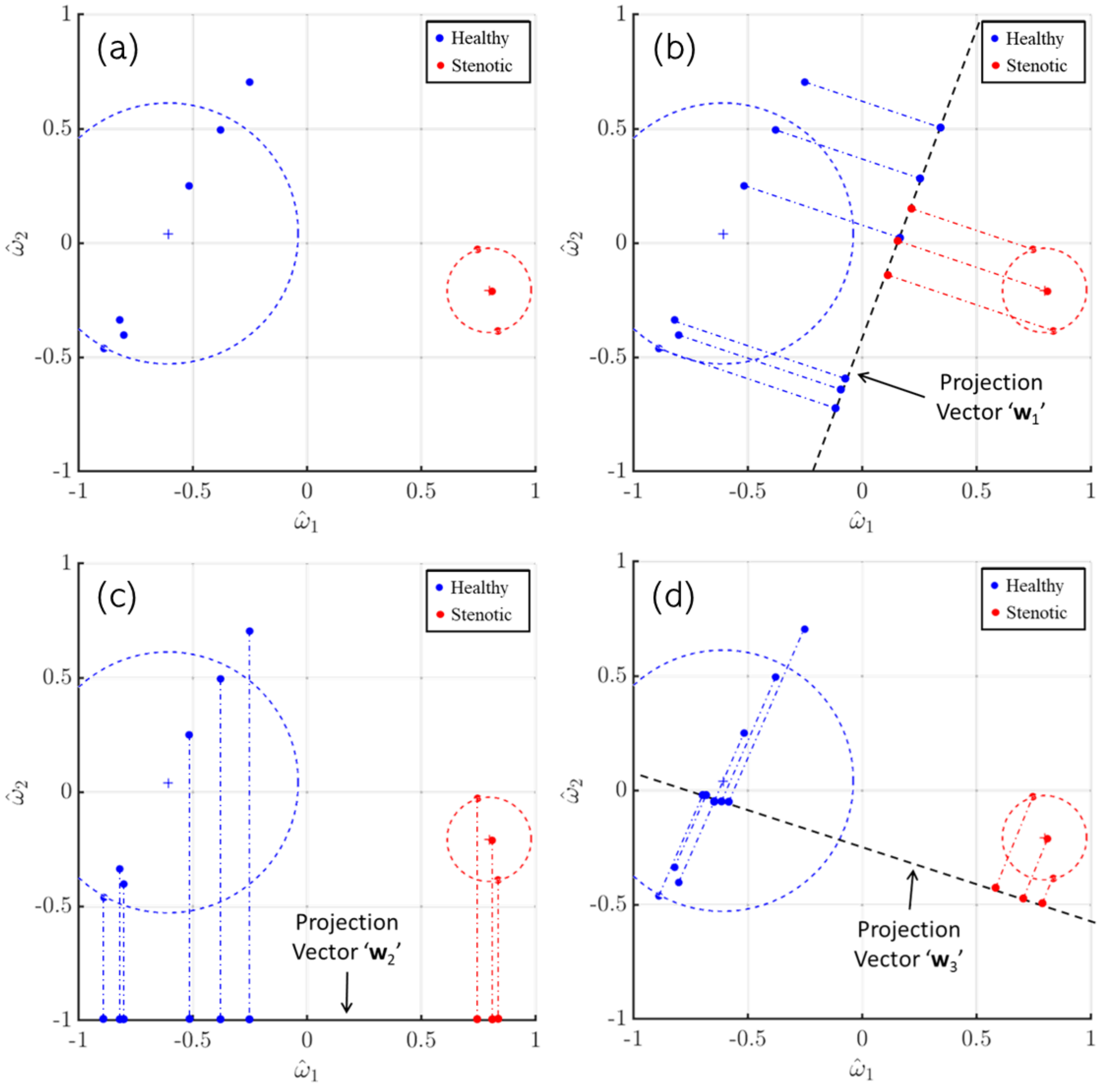

Figure S1: Schematic representation of the linear discriminant analysis procedure using a sample two-dimensional dataset, showing (a) the dataset, and projections of the dataset on vectors (shown in black) resulting in (b) poor, (c) good and (d) optimal classification.

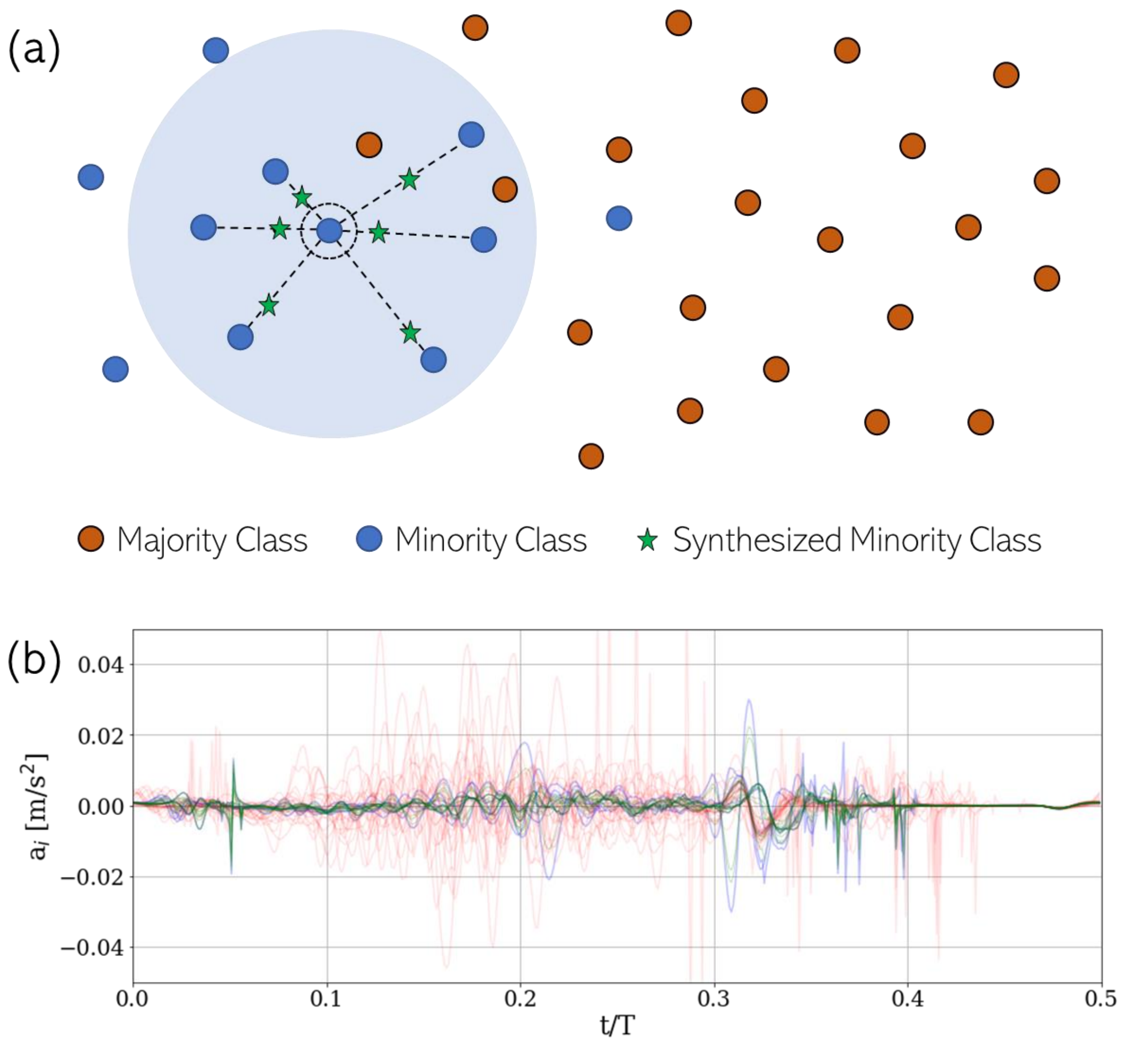

Figure S2: (a) Illustration of SMOTE for a sample two-dimensional dataset. (b) Complete development set showing 29 signals from simulations (blue: Healthy, red: Stenotic) and 15 synthesized Healthy signals (green).

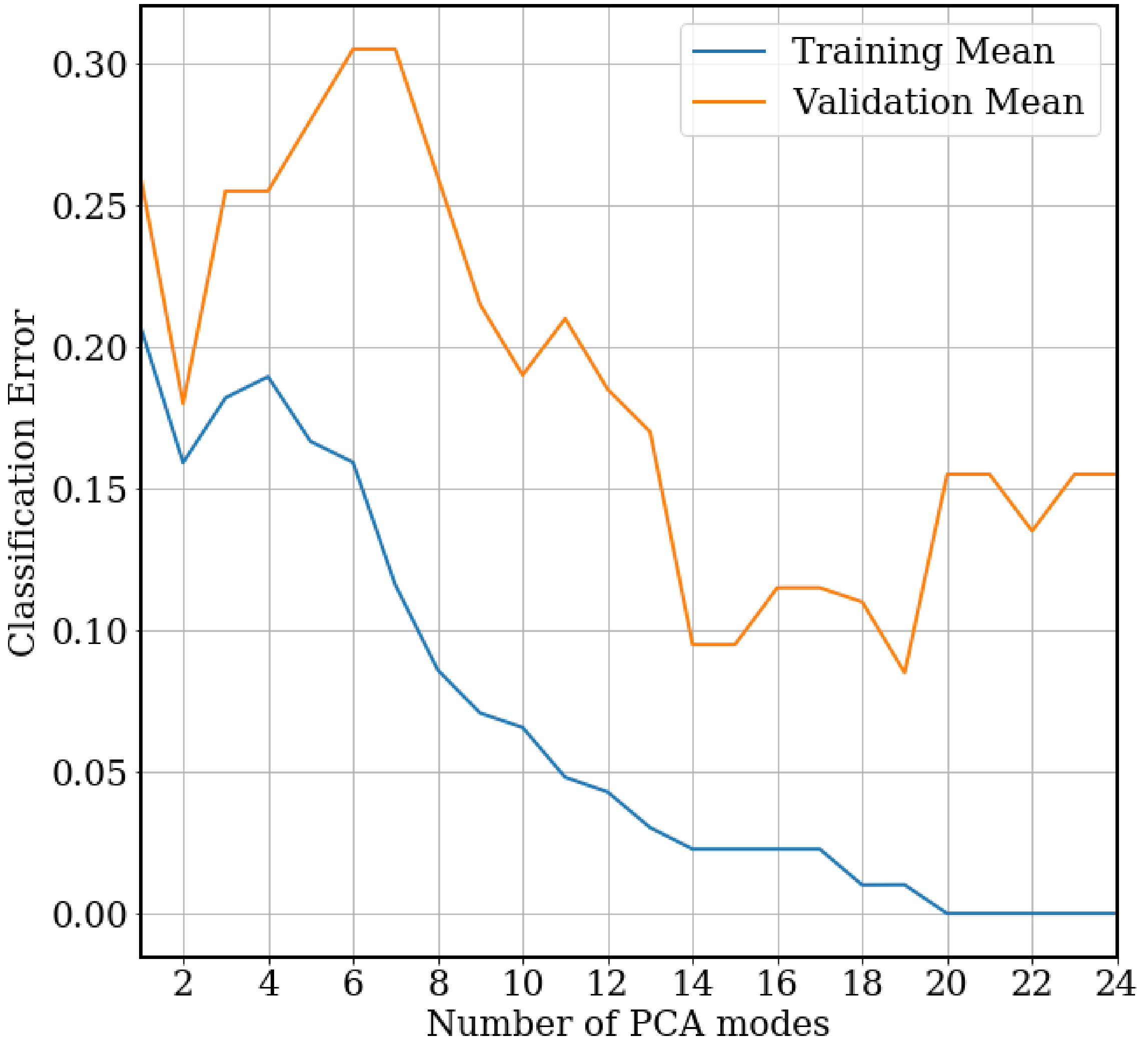

Figure S3: Mean classification error, with varying number of PCA modes, obtained on the training and validation sets.

Tables:

Simulation	Valve State	Simulation Parameter				
		Peak AOA [cm^2]	% Stenosis	Mapping	Stroke Volume [ml]	Flow profile
1	Healthy	4.154	0.00	Linear	60	Centered
2	Healthy	4.154	0.00	Power	60	Centered
3	Healthy	4.154	0.00	Power	72	Centered
4	Healthy	3.739	9.99	Linear	60	Centered
5	Healthy	3.741	9.96	Linear	72	Centered
6	Healthy	3.741	9.96	Linear	60	Eccentric
7	Healthy	3.408	17.97	Power	60	Centered
8	RLM1	3.246	21.87	Linear	60	Eccentric
9	RLM1	3.246	21.87	Linear	60	Centered
10	RLM1	3.322	20.02	Linear	72	Centered
11	RLM1	2.910	29.97	Linear	60	Centered
12	RLM1	3.038	26.87	Power	60	Centered
13	RLM1	2.572	38.08	Power	60	Centered
14	RLM1	2.546	38.73	Power	60	Centered
15	RLM1	2.376	42.80	Power	60	Centered
16	RLM2	2.869	30.93	Linear	60	Centered
17	RLM2	3.024	27.20	Linear	72	Centered
18	RLM2	2.810	32.37	Power	60	Centered
19	RLM2	1.900	54.25	Power	60	Centered
20	RLM2	2.165	47.89	Power	60	Centered
21	RLM2	2.131	48.72	Power	60	Centered
22	RLM2	1.941	53.27	Power	60	Centered
23	RLM2	1.865	55.12	Power	60	Centered
24	RLM3	2.046	50.75	Power	60	Centered
25	RLM3	2.014	51.52	Power	60	Centered
26	RLM3	1.937	53.39	Power	60	Centered
27	RLM3	1.889	54.53	Power	60	Centered
28	RLM3	1.847	55.55	Power	60	Centered
29	RLM3	1.732	58.31	Power	60	Centered

Table S1: Simulation parameters for the development set

Simulation	Valve State	Simulation Parameter				
		Area [cm^2]	% Stenosis	Mapping	Stroke Volume	Flow profile
1	RLM1	2.968	20.66	Power	60	Centered
2	RLM1	2.477	33.78	Power	60	Centered
3	RLM2	2.794	25.31	Power	60	Centered
4	RLM2	2.328	37.77	Power	60	Centered
5	RLM2	2.208	40.96	Power	60	Centered

Table S2: Simulation parameters for the test set
